# Genome-wide linkage mapping of Fusarium crown rot in common wheat (*Triticum aestivum* L.)

**DOI:** 10.3389/fpls.2024.1457437

**Published:** 2024-11-01

**Authors:** Faji Li, Can Guo, Qi Zhao, Weie Wen, Shengnan Zhai, Xinyou Cao, Cheng Liu, Dungong Cheng, Jun Guo, Yan Zi, Aifeng Liu, Jianmin Song, Jianjun Liu, Jindong Liu, Haosheng Li

**Affiliations:** ^1^ Crop Research Institute, National Engineering Laboratory for Wheat and Maize, National Key Laboratory of Wheat Improvement, Key Laboratory of Wheat Biology and Genetic Improvement in the Northern Yellow-Huai Rivers Valley of Ministry of Agriculture and Rural Affairs, Shandong Academy of Agricultural Sciences, Jinan, China; ^2^ Shangqiu Academy of Agriculture and Forestry Sciences, Shangqiu, China; ^3^ Collage of Life Science, Yantai University, Yantai, China; ^4^ Department of Cell Biology, Zunyi Medical University, Zunyi, Guizhou, China; ^5^ Institute of Crop Sciences, National Wheat Improvement Center, Chinese Academy of Agricultural Sciences (CAAS), Beijing, China

**Keywords:** common wheat, Fusarium crown rot (FCR), molecular marker-assisted selection, quantitative trait Loci (QTL), kompetitive allele-specific PCR (KASP)

## Abstract

**Introduction:**

Fusarium crown rot (FCR) is a severe soil-borne disease that affects wheat globally and leads to significant yield reductions. Identifying the loci associated with resistance to FCR and developing corresponding markers are essential for the breeding of resistant wheat varieties.

**Methods:**

In this study, we evaluated the resistance to FCR in a recombinant inbred line (RIL) population originating from Gaocheng 8901 and Zhoumai 16 across four environments. The RILs and their parents were genotyped using a wheat 90K singlenucleotide polymorphism (SNP) array.

**Results:**

We identified a total of five quantitative trait loci (QTLs) related to FCR resistance: *QFCR.caas-3AL*, *QFCR.caas-3DL*, *QFCR.caas-5BL*, *QFCR.caas-6BS*, and *QFCR.caas-7DS*. These QTLs accounted for 4.6% to 12.8% of the phenotypic variance. Notably, *QFCR.caas-5BL* and *QFCR.caas-6BS* had been previously detected, whereas *QFCR.caas-3AL*, *QFCR.caas-3DL*, and *QFCR.caas-7DS* are novel loci. The favorable alleles of *QFCR.caas-3DL* and *QFCR.caas-5BL* were contributed by Zhoumai 16, while the favorable alleles for *QFCR.caas-3AL*, *QFCR.caas-6BS*, and *QFCR.caas-7DS* originated from Gaocheng 8901. Additionally, this study identified seven candidate genes that encode disease resistance proteins, the BTB/POZ domains, peroxidase activity, and leucine-rich repeat receptor-like protein kinase. Furthermore, we developed and validated two kompetitive allele-specific PCR (KASP) markers, *Kasp_3AL_FCR* (*QFCR.caas-3AL*) and *Kasp_5BL_FCR (QFCR.caas-5BL)*, in a natural population of 202 wheat varieties.

**Discussion:**

This study contributes new genetic insights and provides new stable loci and available KASP markers for breeding to enhance FCR resistance in common wheat.

## Introduction

Wheat production is seriously affected by biotic diseases such as stripe rust, powdery mildew, and Fusarium crown rot (FCR). It is crucial in modern wheat breeding to breed higher-yielding and more stable accessions under high disease stress. FCR, e.g. foot rot or root rot, caused by *Fusarium pseudograminearum* and *Fusarium culmorum* (Beccari et al., 2011; Rasheed et al., 2016; Winter et al., 2019; Gao et al., 2023; Feng et al., 2023), is one of the most important diseases affecting wheat and barley and causes significant yield losses, particularly under drought stress (Winter et al., 2019; Gao et al., 2023). The FCR pathogen infects seedlings early in crop development through hyphal penetration, and the infection is facilitated by surface soil moisture (Pariyar et al., 2020; Bozoğlu et al., 2022; Feng et al., 2023; Gao et al., 2023). The most pronounced symptoms of FCR in infected plants are usually characterized by the browning of the coleoptile, leaf, leaf sheath, and stem base, which becomes evident after planting, and can result in plants utterly devoid of grains or possessing shriveled grains (Feng et al., 2023).

FCR resistance has been evaluated in a wide range of wheat germplasms (Duan et al., 2022; Buster et al., 2023; Gao et al., 2023). Previous studies have shown that wheat accessions with a high FCR resistance are rare. Nearly all currently popular cultivars in the Yellow and Huai wheat region are either moderately or highly susceptible to FCR. Although FCR can be controlled by cultivation or chemical approaches, planting FCR-resistant wheat cultivars is the most effective, economical, and environmentally friendly way (Rasheed et al., 2016; Rahman et al., 2021; Zheng et al., 2022; Xiong et al., 2023). Previous wheat resistance breeding mainly focused on stripe rust, leaf rust, and powdery mildew, while FCR resistance breeding has seldom been reported.

Wheat FCR resistance is a typical quantitative trait and is determined by multiple minor genes (Liu and Ogbonnaya, 2015; Li M. et al., 2022; Li J. et al., 2022). Currently, knowledge of FCR resistance loci with higher effects and available markers is still limited. Thus, it is imperative to identify the significant loci/genes associated with FCR resistance (Lin et al., 2022; Hou et al., 2023; Mao et al., 2023; Lv et al., 2023). Currently, with the development of the re-sequencing and single-nucleotide polymorphism (SNP) assays (Wang et al., 2014), genome-wide linkage mapping and genome-wide association analysis (GWAS) have been widely applied to uncover the genetic basis of complex agronomical traits (Maccaferri et al., 2016). Over the past two decades, numerous quantitative trait loci (QTLs) for FCR resistance have been identified and are distributed across 13 of the 21 chromosomes (Erginbas-Orakci et al., 2018; Yang et al., 2019; Jin et al., 2020; Pariyar et al., 2020; Su et al., 2021; Gao et al., 2023; Hou et al., 2023; Mao et al., 2023; Lin et al., 2023; Lv et al., 2023; Wang et al., 2024). One of the most effective FCR-resistant QTLs is located on chromosome 3BL which originated from the ‘CSCR6/Lang’ recombinant inbred line (RIL) population and explains 49% of the phenotypic variation (Yang et al., 2019). Additionally, a locus on chromosome 4B was identified from a cross of ‘Kukri/Janz’ near the dwarfing gene *Rht1* (Malosetti et al., 2021). However, there are several issues: (1) most loci have not been validated in other populations, so it is uncertain whether they are widely present or applicable; (2) some loci are derived from GWAS analysis and may be false positives; (3) most of the discovered loci have low phenotypic effects and may not meet breeding requirements, necessitating further exploration of new loci; and (4) some studies are based on landraces or wild species, which are may not be suitable for or conducive to breeding applications.

Due to climate change, FCR has become one of the more serious fungal diseases in the Yellow and Huai Valley Facultative Wheat Region in China. Breeding FCR-resistant cultivars is both important and urgent. In this study, we conducted genome-wide linkage mapping for FCR resistance using the wheat 90K assays in the Gaocheng8901/Zhoumai16 RIL population. The main goal of this study was to gain insight into the genetic basis of FCR resistance, identify new FCR resistance loci/genes, and develop available kompetitive allele-specific PCR (KASP) markers for breeding.

## Materials and methods

### Plant materials and field trials

**Figure 1:**
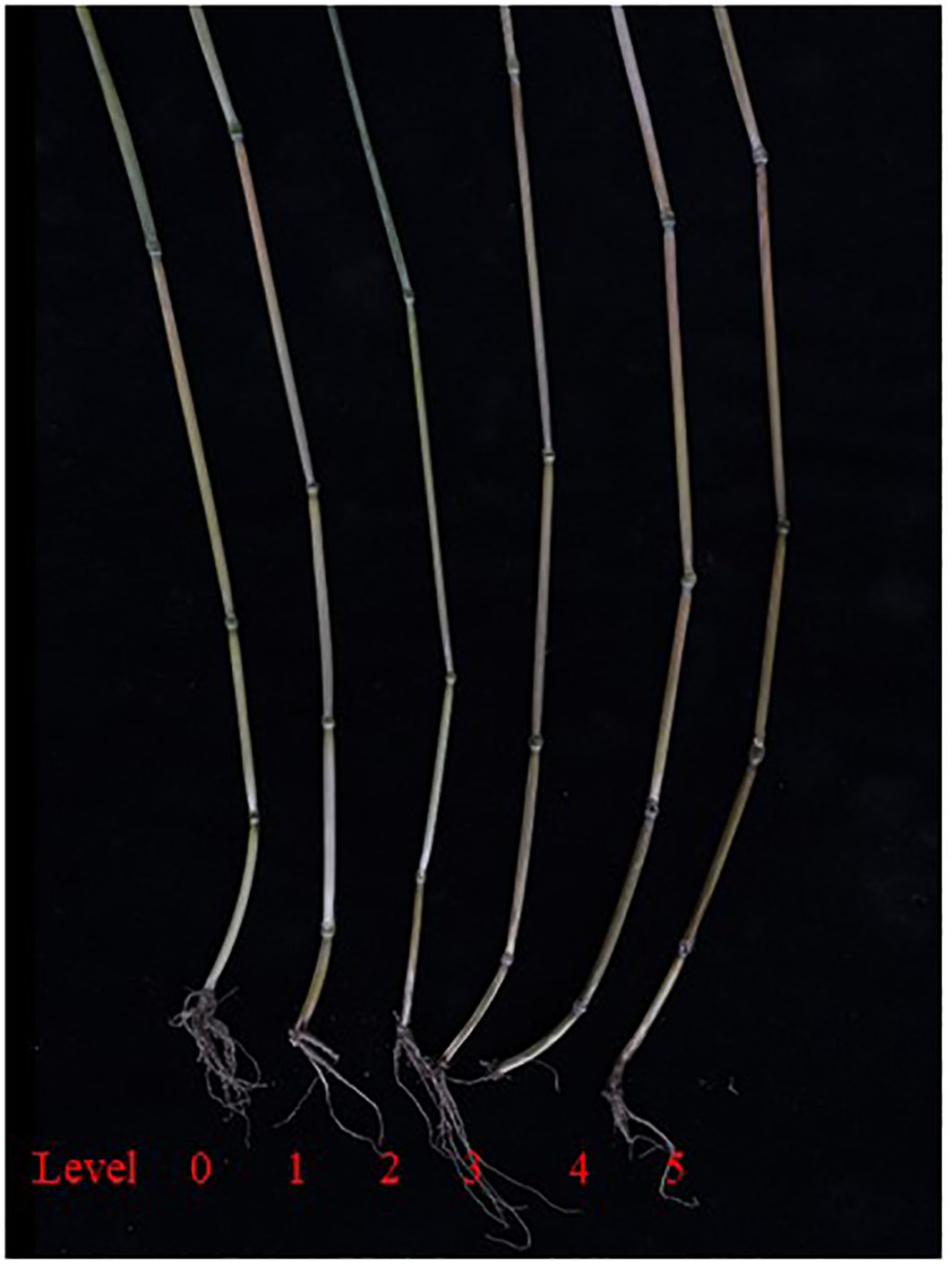
The FCR resistance level in common wheat.

The 176 RILs originated from a Gaocheng 8901/Zhoumai 16 cross (**Figure 1**). Although both Gaocheng 8901 and Zhoumai 16 are moderately susceptible to FCR, Gaocheng 8901 showed higher FCR resistance than Zhoumai 16. A panel comprising 202 varieties, primarily from the Yellow and Huai River Valley Facultative Wheat Region in China, was used to validate the effects of the KASP markers. The 176 RILs and the association panel were evaluated for the FCR index in Jinan and Dezhou, Shandong province, during the 2020-2021 and 2021-2022 cropping seasons. The field trials were designed using a completely randomized block design according to Li et al. (2018), and included three replications at all locations. Each plot consisted of six rows, each 3.0 m long, 1.2 m wide, and spaced 0.2 m apart, with 50 grains sowed per row. The field trials were managed according to local agricultural practices.

### Evaluation of the FCR index

The experiments were conducted in fields heavily threatened with a uniform incidence of disease. The soil, characterized by medium fertility, was prepared by deep plowing and rotary tillage before sowing. Investigations took place at the wheat wax ripeness stage, focusing primarily on assessing the white spike rate and the disease index. For each row of varieties, the number of white spikes and the total number of spikes were counted to determine the white spike rate. In each row, three points were selected, and 20 individual stems were collected for analysis. Disease severity was graded according to established field trial standards at the mature plant stage, and the disease index was calculated.

The standards for grading disease severity at the wax ripeness stage in field trials are as follows: level 0: no symptoms of browning in the innermost leaf sheath or the entire culm; level 1: significant browning in the innermost leaf sheath of the aboveground part, but no browning or rotting in the first internode; level 2: browning and rotting symptoms in the first internode of the aboveground part; level 3: browning and rotting symptoms in the second internode of the aboveground part; level 4: browning and rotting symptoms in the third internode of the aboveground part; level 5: disease spots extend beyond the third internode, or the plant exhibits white spikes or no spikes due to disease onset.

The disease index was calculated using the formula (Yang et al., 2015):


Disease Index = (∑ (number of plants at each disease level × representative value for each level)) / (total number of plants surveyed × representative value for the most severe disease level) × 100

### Statistical analyses

Analysis of variance (ANOVA) was conducted using PROC GLM in SAS v9.0. To evaluate the size and significance of the effects, the environment, line, environment-line interactions, and replicates were considered random effects. Broad-sense heritability (*H*_b_^2^) for wheat FCR was calculated according to *H*_b_^2^ = σ^2g^/(σ^2g^+σ^2ge^/e+σ^2ϵ^/re), where σ^2g^, σ^2ge^, and σ^2ϵ^ represent the estimates of variance of line, line × environment interaction, and residual error, respectively (Nyquist et al., 1991). Finally, *r* and *e* denote the number of replicates and environments, respectively.

### Linkage mapping

The wheat 90K SNP array was employed to genotype the RILs and their parents. The BIN function in IciMapping v4.2 (Meng et al., 2015) was used to select the backbone markers according to Wen et al. (2017) and Li et al. (2018). Linkage groups were constructed using JoinMap v4.0 (http://www.kyazma.nl/index.php/JoinMap/). Linkage analysis was performed via the ICIM model in IciMapping v4.2, at a walking speed of 0.1 cM. A logarithm of odds (LOD) threshold of 2.8 was set for identifying significant QTLs, which was derived from 2,000 permutations at a significance level of *P* = 0.05. QTLs identified in two or more environments were deemed stable. Wen et al. (2017) and Li et al. (2018) have reported the Gaocheng8901/Zhoumai 16 high-density linkage map.

### Development and validation of the KASP markers

KASP primers were designed using PolyMarker for the target polymorphic sites. Primer premixes were prepared according to the methodology described by Yang et al. (2020), and the PCR procedure was conducted following the protocol outlined by Li et al. (2021). KlusterCaller™ v2.24.0.11 was utilized to read the different fluorescence signal values and to analyze the genotypes. Successfully converted KASP markers were used for genotyping and validated using 202 wheat cultivars.

### Candidate gene prediction

To identify candidate genes associated with the identified QTLs for FCR in the Gaocheng8901/Zhoumai16 RIL population, genes situated in the LD (linkage disequilibrium) block region around the peak SNP (± 3.0 Mb, according to prior LD decay analysis) for each QTL were annotated using IWGSC v1.1 (https://wheat.pw.usda.gov/GG3/). Genes that were not hypothetical proteins, transposable elements, or retro-transposable elements, and which had SNPs in the coding region, were regarded as candidate genes. The resistance mechanism of FCR in wheat is complex and likely involves the following three aspects: enhancing FCR resistance by regulating the expression of defense genes such as *TaMPK3*, *TaPR1*, and *TaChitinase*; detoxifying fungal toxins through glycosylation or opening of epoxy groups to inhibit pathogen infection and colonization; and regulating cell wall thickness to increase physical resistance. Some plant hormones and stress tolerance genes may also be related to FCR. Therefore, when selecting candidate genes, special attention should be paid to the aforementioned relevant annotated genes. In addition, gene expression profiles were examined using expVIP (http://www.wheat-expression.com/), and genes that were specifically and highly expressed in grains, spikes, or stems were selected.

## Results

### Phenotypic analysis

The FCR incidences in Gaocheng8901, Zhoumai16, and the RILs were phenotyped across four environments (**Supplementary Figure S1**, **Supplementary Table S1**). The FCR index in the 176 RILs was continuously distributed, ranging from 24.9% to 51.1% in Jinan 2021 (mean FCR index of 37.6%, standard error (SD) of 5.0%, and coefficient of variation (CV) of 13.2%), 16.9% to 41.3% in Dezhou 2021 (mean FCR index of 26.9%, SD of 4.9%, and CV of 18.1%), 19.1% to 47.1% in Jinan 2022 (mean FCR index of 31.6%, SD of 5.3%, and CV of 16.7%), and 19.6% to 42.1% in Dezhou 2022 (mean FCR index of 32.2%, SD of 4.2%, and CV of 13.2%). The FCR incidence rates for the RIL population were significantly correlated (*r*=0.43-0.84, *P*<0.01) across the four environments (**Supplementary Table S2**), demonstrating a high *H*_b_^2^ (0.74). The ANOVA showed considerable significance (*P*<0.001) among genotypes, environments, and genotype × environment interactions (**Table 1**).

**Table 1:** ANOVA of the FCR index in the Gaocheng 8901/Zhoumai 16 RIL population.

Source of variation	DF	Sum of square	Mean square	*F-value*
Replicate	8	65	8	0.9**
Environment	3	30496	10165	1137.5**
Genotype	175	32661	186	20.8**
Genotype×Environment	525	16978	32	3.6**
Error	1400			

**significant at *P* < 0.001.

*H*_b_^2^: 0.74.

### Linkage map construction

In the Gaocheng8901/Zhoumai16 RIL population, 3,284 skeleton markers were selected to construct a genetic linkage map that comprised 30 linkage groups with a total genetic distance of 3,130.3 cM. The B genome had the largest number of skeleton markers, the longest total genetic linkage map, and the highest average marker density on chromosomes. In contrast, the D genome contained the fewest skeleton markers, the shortest genetic linkage distance, and the lowest marker density. Across the entire genome, the average marker density was 1.04 markers/cM. We have published the Gaocheng 8901/Zhoumai 16 RIL population linkage map in previous studies, including all the details of the marker and linkage groups (Wen et al., 2017; Li et al., 2018).

### Loci for FCR resistance

**Figure 2:**
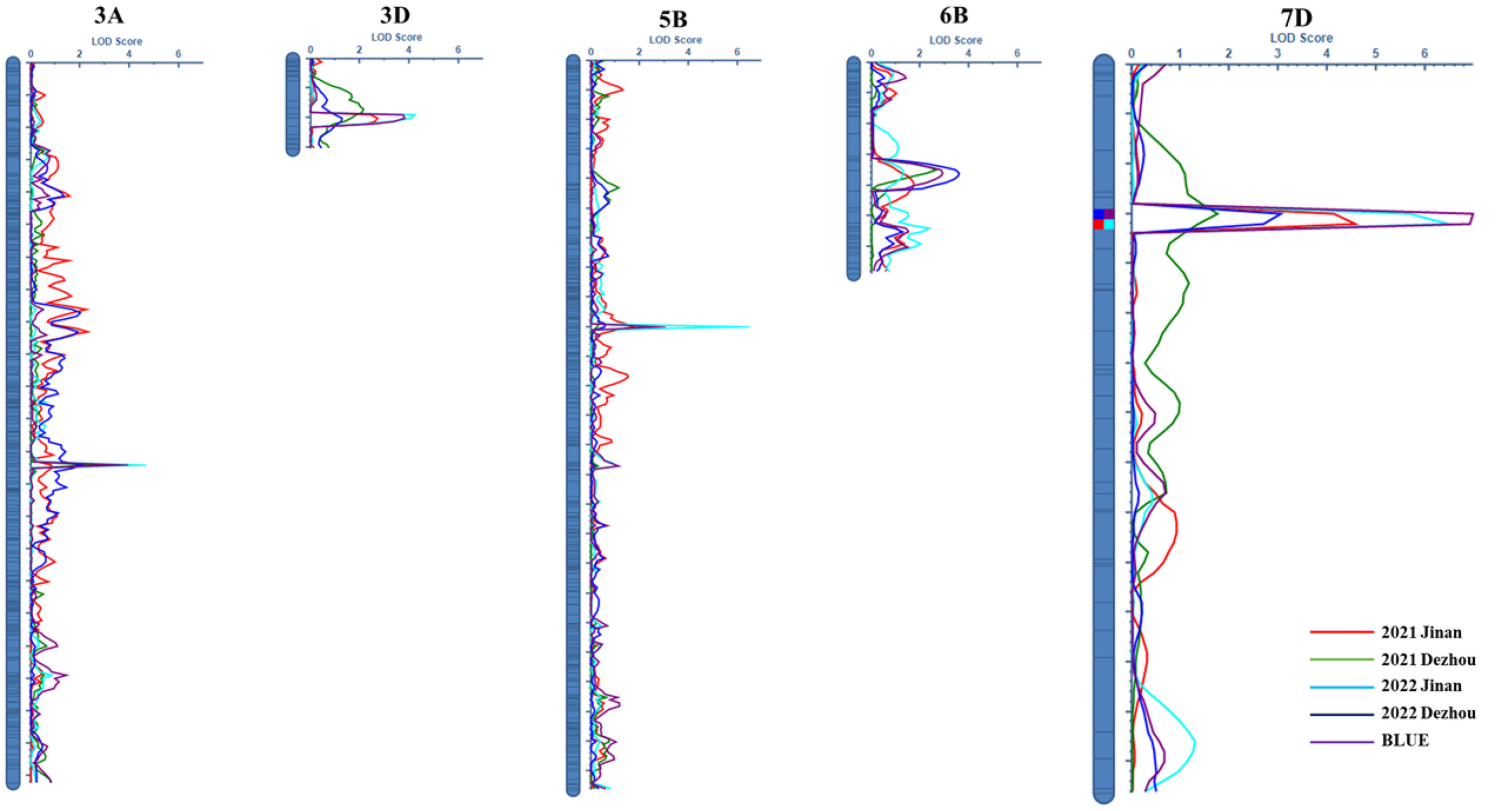
LOD contours obtained using composite interval mapping of the QTLs for the FCR index in the Gaocheng 8901/Zhoumai 16 RIL population.

Five QTLs for FCR resistance were distributed on chromosomes 3AL, 3DL, 5BL, 6BS, and 7DS, namely, *QFCR.caas-3AL*, *QFCR.caas-3DL*, *QFCR.caas-5BL*, *QFCR.caas-6BS*, and *QFCR.caas-7DS*, respectively (**Figure 2**, **Table 2**). *QFCR.caas-3AL* was detected in Jinan 2021, Jinan 2022, and the best linear unbiased estimator (BLUE), explaining 6.0%-6.9% of the phenotypic variances, with additive effects ranging from 1.08 to 1.20. *QFCR.caas-3DL*, identified in Jinan 2022, Dezhou 2022, and the BLUE, contributed 6.5%-7.1% of the phenotypic variances, with additive effects ranging from -1.04 to -1.21. *QFCR.caas-5BL* was detected in Jinan 2022, Dezhou 2022, and the BLUE, explaining 4.6%-9.7% of the phenotypic variances, with additive effects from -0.89 to -1.43. *QFCR.caas-6BS*, identified in Dezhou 2021, Dezhou 2022, and the BLUE, accounted for 5.3%-8.7% of the phenotypic variances, with additive effects ranging from 0.98 to 1.44. *QFCR.caas-7DS* was identified in Jinan 2021, Jinan 2022, Dezhou 2022, and the BLUE, explaining 6.6%-12.8% of the phenotypic variances, with additive effects ranging from 0.27 to 1.57. The resistance alleles of *QFCR.caas-3DL* and *QFCR.caas-5BL* were from Zhoumai16, whereas those of *QFCR.caas-3AL*, *QFCR.caas-6BS*, and *QFCR.caas-7DS* were contributed by Gaocheng8901.

**Table 2:** Composite interval mapping of QTLs for the FCR index in the Gaocheng 8901/Zhoumai 16 RIL population.

Chromosome	Marker interval	Genetic position (cM)	Physical (Mb)	PVE	LOD	Add	Confidence interval
*QFCR.caas-3AL*	*BS00076772_51-BS00023222_51*	12	711.2-714.6	6.0-6.9	2.6-4.2	1.08-1.20	18.5-21.5
*QFCR.caas-3DL*	*RAC875_c44290_511-BS00094456_51*	14	636.5-648.7	6.5-7.1	3.6-4.3	-1.04–1.21	123.5-124.5
*QFCR.caas-5BL*	*IACX5818-Tdurum_contig13219_371*	23	442.3-443.2	4.6-9.7	2.7-6.2	-0.89–1.43	89.5-90.5
*QFCR.caas-6BS*	*Tdurum_contig47020_173-Tdurum_contig11539_81*	30	42.3-45.3	5.3-8.7	2.6-3.5	0.98-1.44	33.5-39.5
*QFCR.caas-7DS*	*BobWhite_rep_c65034_450-RAC875_rep_c113244_113*	34	90.6-91.9	6.6-12.8	3.0-7.0	0.27-1.57	14.5-16.5

### Identified candidate genes

A total of seven candidate genes for wheat FCR resistance were identified (**Table 3**) based on physical location, functional annotation, and expression information from the expVIP database. The seven candidate genes were all expressed in the roots, stems, and spikes of the wheat. *TraesCS3A01G479300* and *TraesCS3A01G486800* were the candidate genes for *QFCR.caas-3AL* and were encoded by the disease resistance protein RPP13 and a BTB/POZ domain-containing protein, respectively. *TraesCS5B01G259900*, which encodes the NBS-LRR disease resistance protein, was identified for *QFCR.caas-5BL*. *TraesCS6B01G063700* encoded a peroxidase and was identified for *QFCR.caas-6BS*. *TraesCS6B01G064900* was the candidate gene for *QFCR.caas-6BS* and encoded the disease-resistance protein RPM1. Both *TraesCS7D01G140100* and *TraesCS7D01G145100* were located at the genetic interval of *QFCR.caas-7DS* and encoded the NBS-LRR disease resistance protein and a leucine-rich repeat receptor-like protein kinase, respectively.

**Table 3:** The candidate genes for FCR resistance identified in this study.

QTL	Candidate gene	Start (bp)	End (bp)	Annotation
*QFCR.caas-3AL*	*TraesCS3A01G479300*	711040605	711041129	Disease-resistance protein RPP13
*QFCR.caas-3AL*	*TraesCS3A01G486800*	714768191	714769599	BTB/POZ domain-containing protein
*QFCR.caas-5BL*	*TraesCS5B01G259900*	442379290	442382389	NBS-LRR disease-resistance protein
*QFCR.caas-6BS*	*TraesCS6B01G063700*	42230050	42231754	Peroxidase
*QFCR.caas-6BS*	*TraesCS6B01G064900*	43080180	43086099	Disease-resistance protein RPM1
*QFCR.caas-7DS*	*TraesCS7D01G140100*	89530263	89533017	NBS-LRR disease-resistance protein,
*QFCR.caas-7DS*	*TraesCS7D01G145100*	92589418	92592854	Leucine-rich repeat receptor-like protein kinase

### Validation of the KASP markers

**Figure 3:**
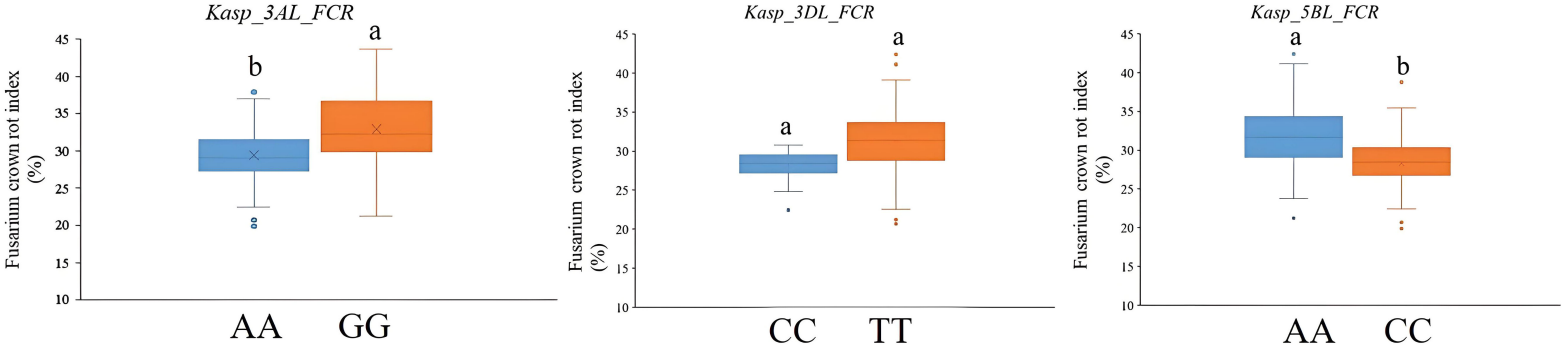
Validation of *Kasp_3AL_FCR, Kasp_3DL_FCR*, and *Kasp_5BL_FCR* in the panel of 202 wheat cultivars from the Huang-Huai River Valleys region. Different letters indicate significant differences at the *P <*0.05 level.

All five QTLs for FCR resistance were used to develop the KASP markers. However, the KASP marker for *QFCR.caas-6BS* was chromosome unspecific and could not be effectively used in wheat breeding. Moreover, although efforts were made to develop a KASP marker for *QFCR.caas-7DS*, it failed to effectively differentiate between the two parental genotypes in the RIL population, leading to inconclusive results. Consequently, three KASP markers, *Kasp_3AL_FCR* corresponding to *BS00023222_51* (located on *QFCR.caas-3AL*), *Kasp_3DL_FCR* corresponding to *BS00094456_51* (located on *QFCR.caas-3DL*), and *Kasp_5BL_FCR* corresponding to *Tdurum_contig13219_371* (located on *QFCR.caas-5BL*) were successfully developed (**Table 4**). To verify the effectiveness of these three KASP markers, a total of 202 diverse cultivars were used. For *Kasp_3AL_FCR*, the favorable allele (AA, accounting for 58.4%, mean FCR index: 29.4%) exhibited a lower FCR index compared to the unfavorable allele (GG, 34.6%, FCR index: 32.9%) which was significant at the *P*=0.05 level. For *Kasp_3DL_FCR*, the favorable allele (CC, accounting for 20.7%, mean FCR index: 28.1%) exhibited a lower FCR index compared to the unfavorable allele (TT, 59.9%, FCR index: 31.3%), but the difference was not significant at the *P*=0.05 level. For *Kasp_5BL_FCR*, the favorable allele (CC, 32.7%, FCR index: 28.0%) showed a lower FCR index compared to the unfavorable allele (AA, 57.4%, FCR index: 32.0%) at the *P*=0.05 level (**Figure 3**, **Supplementary Table S3**).

**Table 4:** The developed and validated KASP markers in this study that can be used to improve FCR resistance.

Locus	Marker	Kasp Marker	Primer	Sequence
*QFCR.caas-3AL*	*BS00023222_51*	*Kasp_3AL_FCR*	FAM	GAAGGGGACCAAGTTAATGCTAAGCACCTACGACGACATGT
			HEX	GAAGCCCGAAGTCAACGGATTAAGCACCTACGACGACATGC
			Common	AGAGGGAACGAGCAGGGCTAATAGGGCGGCTCCTGCTA
*QFCR.caas-3DL*	*BS00094456_51*	*Kasp_3DL_FCR*	FAM	GAAGGGGACCAAGTTAATGCTCTCTTCCTTGGGATGGGA
			HEX	GAAGCCCGAAGTCAACGGATTGCTCTTCCTTGGGATGGGG
			Common	AGAGGGAACGAGCAGGGCAGGAATGACCATGCCACAG
*QFCR.caas-5BL*	*Tdurum_contig13219_371*	*Kasp_5BL_FCR*	FAM	GAAGGGGACCAAGTTAATGCTGGATGCCATCTTTGTCGCT
			HEX	GAAGCCCGAAGTCAACGGATTGGATGCCATCTTTGTCGCG
			Common	AGAGGGAACGAGCAGGGCTGCATGATCGAGAAGTATAAGAGTT

FAM: GAAGGGGACCAAGTTAATGCT.

HEX: GAAGGTCGGAGTCAACGGATT.

Common: taatagggcggctcctgcta.

The underlined values mean the FAM, HEX and Common primers.

## Discussion

FCR has caused severe damage to wheat production in China (Su et al., 2021). However, FCR was not considered a major threat to wheat production until 2015. Yang et al. (2019) found that nearly 83% of the 234 wheat cultivars were susceptible to FCR, with only seven resistant accessions identified. Currently, progress in research on wheat FCR is slow, with limited resistant germplasm resources that are unable to meet the current breeding needs for disease resistance, and a lack of directly applicable resistance genes and molecular markers (Su et al., 2021; Lv et al., 2023). FCR resistance is a typical complex quantitative trait. Environment and genotype have a significant impact on the severity of FCR. In this study, *QFCR.caas-3AL*, *QFCR.caas-3DL*, *QFCR.caas-5BL*, *QFCR.caas-6BS*, and *QFCR.caas-7DS* were identified using the Gaocheng8901/Zhoumai16 RIL population and they explained 6.0%-6.9%, 6.5%-7.1%, 4.6%-9.7%, 5.3%-8.7%, and 6.6%-12.8% of the phenotypic variances, respectively.

Until now, over 40 loci for FCR have been reported in the wheat genome and are evenly distributed in the A, B, and D genomes (Erginbas-Orakci et al., 2018; Yang et al., 2019; Jin et al., 2020; Pariyar et al., 2020; Su et al., 2021; Singh et al., 2022; Gao et al., 2023; Hou et al., 2023; Mao et al., 2023; Lin et al., 2023; Lv et al., 2023; Wang et al., 2024; http://wheatqtldb.net/). Jin et al. (2020) reported eight seedling FCR resistance loci on chromosomes 1BS, 1DS, 2AL, 5AL, 5DS, 5DL, 6BS, and 7BL. Li et al. (2024) identified seven loci distributed on chromosomes 2B, 3A, 3D, 4A, 7A, and 7B for FCR resistance in 223 wheat accessions. Hou et al. (2023) reported 21 FCR resistance loci on chromosomes 1A, 1B, 2B, 2D, 3B, 3D, 4B, 5A, 5B, 7A, and 7B in 361 Chinese wheat landraces. Pariyar et al. (2020) reported 15 FCR resistance loci with one stable QTL on 3BS near the FHB-resistant *Fhb1*. We identified *QFCR.caas-3AL* at 711.2-714.6 Mb on 3AL, different from the locus identified by Pariyar et al. (2020) (68.3 Mb) and *Qfcr.cau.3A* (746.6 Mb) detected by Ma et al. (2020). *QFCR.caas-3DL* at 636.5-648.7 Mb on the 3DL chromosome is different from the locus identified by Pariyar et al. (2020) (605.3 MB) and *Qfcr.sicau.3D-1* (5.30 Mb) by Hou et al. (2023). In addition, we identified a locus for FCR resistance (*QFCR.caas-5BL*, 442.3-443.2 Mb) on 5BL, which nearly aligns with the locus (457.2 Mb MB) identified by Pariyar et al. (2020), *Qfcr.sicau.5B-1* (76.60 Mb) identified by Hou et al. (2023), and the locus at 514-546 Mb identified by Gurung et al. (2014) on 5B. In addition, *QFCR.caas-6BS (*42.3-45.3 Mb) is different from the loci identified by Grung et al. (2014) (542.0 Mb), Ahirwar et al. (2018) (11.3 Mb), Yang et al. (2019) (534.5 Mb), and Rahman et al. (2020) (663.2 Mb). However, *QFCR.caas-6BS (*42.3-45.3 Mb) overlapped with the loci identified by Jin et al. (2020) (64.3 Mb) and Pariyar et al. (2020) (74.0 Mb). Until now, no locus for FCR was identified on chromosome 7DL. Thus, *QFCR.caas-3AL*, *QFCR.caas-3DL*, and *QFCR.caas-7DS* may be novel.

The results from wheat and barley indicated that multiple FCR QTLs exhibited significant interactions with nearby plant height (PH) or heading date (HD) QTLs that were also present in the same mapping populations (Zheng et al., 2017; Hou et al., 2023; Jin et al., 2020; Li et al., 2024). We have identified six loci for HD and named them *QHD.caas-1BL* (369.2Mb), *QHD.caas-1DL.2* (488.6 Mb), *QHD.caas-2AL* (350.7 Mb), *QHD.caas-5AL* (478.9 Mb), *QHD.caas-7BS* (56.9 Mb), and *QHD.caas-7DL* (426.7 Mb). In addition, we have reported six loci for PH in the Gaocheng8901/Zhoumai16 RIL population and named them *QPH.caas-3AS* (45.6 Mb), *QPH.caas-4BS* (25.8 Mb), *QPH.caas-4DS* (25.4 Mb), *QPH.caas-5AL.2* (595.4 Mb), and *QPH.caas-5BL* (532.3Mb) (Li et al., 2018). There was no overlap between these loci with those identified in this study.

### Prediction of candidate genes for FCR

Seven candidate genes for FCR involved in disease resistance, redox reaction, stress tolerance, and signal transduction were identified. These candidate genes were screened according to the following criteria: (1) the genes are located in or adjacent to the physical intervals of QTL identified, (2) they are related to the molecular processes in FCR response, and (3) they may be differentially expressed in grain spikes or stems (**Supplementary Figure S2**).

*TraesCS3A01G479300* for *QFCR.caas-3AL* and *TraesCS6B01G064900* for *QFCR.caas-6BS* encode the disease resistance protein RPP13 and disease resistance protein RPM1, which play vital roles in the reaction of fungus (Powell et al., 2017). *TraesCS3A01G486800* for *QFCR.caas-3AL* encoded the BTB/POZ domain-containing protein, which serves as an adaptor protein for cullin3-based E3 ubiquitin ligase in plants. It is involved in the regulation of various physiological processes including plant growth, disease resistance, fertility, fatty acid metabolism, and abscisic acid (ABA) signaling pathways (Lin et al., 2022; Bashyal et al., 2022). *TraesCS5B01G259900* for *QFCR.caas-5BL*, *TraesCS7D01G140100* for *QFCR.caas-7DS*, and *TraesCS7D01G145100* for *QFCR.caas-7DS* encoded the NBS-LRR disease-resistance protein or leucine-rich repeat receptor-like protein kinase, which contain a nucleotide-binding site (NBS) and a leucine-rich repeat (LRR) structural domain.

The NBS features three highly conserved key motifs, namely *Kinase-1a*, *Kinase-2*, and *Kinase-3a*, which are capable of binding ATP or GTP to obtain the energy subsequently used to defend against pathogens (Sweat and Wolpert, 2007). The LRR is involved in the recognition of pathogen-derived avirulence proteins and the signaling process and is the main reason for the gene-for-gene specificity in the pathogen recognition of resistance genes. The upregulation of CC-NBS-LRR, LRR repeats, and RLK resistance proteins promotes pathogen recognition, triggering signaling cascades mediated by ethylene and particularly calmodulins (Sweat et al., 2007, Manzo et al., 2016; Powell et al., 2017; Jin et al., 2020). *TraesCS6B01G063700* for *QFCR.caas-6BS* encoded peroxidases, which are a class of enzymes found in many living organisms. Peroxidases catalyze the decomposition of hydrogen peroxide (H_2_O_2_) and other organic peroxides (ROOH), and play a crucial role in stress response and fungi pathogen resistance (Zhang et al., 2013; Daroodi and Taheri, 2023).

### Application for FCR resistance in wheat breeding

Selecting FCR-resistant lines in the field presents significant challenges because resistance can only be assessed in mature seeds after harvest, and the results are heavily influenced by environmental conditions. KASP, a uniplex SNP genotyping platform, solves these problems by providing a cost-effective, flexible, and highly accurate method for marker assisted selection (MAS) and fine mapping of genes (Rasheed et al., 2016). This technology allows for more precise identification of resistant lines, thereby improving the efficiency of breeding programs aimed at combating FCR. In this study, *Kasp_3AL_FCR* (*QFCR.caas-3AL*) and *Kasp_5BL_FCR* (*QFCR.caas-5BL*) were successfully developed and validated in 202 varieties. Thus, the stable FCR resistance QTLs and KASP markers could be used for MAS breeding. Several accessions possessing a high number of resistance alleles and outstanding agronomic traits, such as Shi4185, Heshangtou, Gaocheng8901, Lumai15, Youzimai, and Lovrin10, could be good parental lines in wheat breeding.

## Conclusion

In the present study, five stable FCR resistance QTLs were identified and each explained 4.6%-12.8% of the phenotypic variance explained (PVE), respectively. Seven candidate genes located at the genetic interval of stable FCR resistance QTLs were identified for FCR resistance. Additionally, two KASP markers, *Kasp_3AL_FCR* and *Kasp_5BL_FCR*, were developed and validated in 202 wheat accessions. The new FCR resistance QTLs, available KASP markers, and highly resistant varieties can be used to enhance FCR resistance breeding in wheat.

## Data Availability

The original contributions presented in the study are included in the article/**Supplementary Material**. Further inquiries can be directed to the corresponding author.
