# Corrigendum: Genome-wide linkage mapping of Fusarium crown rot in common wheat (*Triticum aestivum* L.)

**DOI:** 10.3389/fpls.2025.1563575

**Published:** 2025-02-06

**Authors:** Faji Li, Can Guo, Qi Zhao, Weie Wen, Shengnan Zhai, Xinyou Cao, Cheng Liu, Dungong Cheng, Jun Guo, Yan Zi, Aifeng Liu, Jianmin Song, Jianjun Liu, Jindong Liu, Haosheng Li

**Affiliations:** ^1^ Crop Research Institute, National Engineering Laboratory for Wheat and Maize, National Key Laboratory of Wheat Improvement, Key Laboratory of Wheat Biology and Genetic Improvement in the Northern Yellow-Huai Rivers Valley of Ministry of Agriculture and Rural Affairs, Shandong Academy of Agricultural Sciences, Jinan, China; ^2^ Shangqiu Academy of Agriculture and Forestry Sciences, Shangqiu, China; ^3^ Collage of Life Science, Yantai University, Yantai, China; ^4^ Department of Cell Biology, Zunyi Medical University, Guizhou, Zunyi, China; ^5^ Institute of Crop Sciences, National Wheat Improvement Center, Chinese Academy of Agricultural Sciences (CAAS), Beijing, China

**Keywords:** common wheat, Fusarium crown rot (FCR), molecular marker-assisted selection, quantitative trait Loci (QTL), kompetitive allele-specific PCR (KASP)

In the published article, there was an error. A correction has been made to the **Abstract**.

This paragraph previously stated:

“**Introduction:** Powdery mildew (PM) poses an extreme threat to wheat yields and quality z. [Methods] In this study, 262 recombinant inbred lines (RILs) of Doumai and Shi 4185 cross were used to map PM resistance genes across four environments. A high-density genetic linkage map of the Doumai/Shi 4185 RIL population was constructed using the wheat Illumina iSelect 90K single nucleotide polymorphism (SNP) array.

**Results:** In total, four stable quantitative trait loci (QTLs) for PM resistance, *QPm.caas-2AS*, *QPm.caas-4AS*, *QPm.caas-4BL*, and *QPm.caas-6BS*, were detected and explained 5.6%–15.6% of the phenotypic variances. Doumai contributed all the resistance alleles of *QPm.caas-2AS*, *QPm.caas-4AS*, *QPm.caas-4BL*, and *QPm.caas-6BS*. Among these, *QPm.caas-4AS* and *QPm.caas-6BS* overlapped with the previously reported loci, whereas *QPm.caas-2AS* and *QPm.caas-4BL* are potentially novel. Additionally, six high-confidence genes encoding the NBS-LRR-like resistance protein, disease resistance protein family, and calcium/calmodulin-dependent serine/threonine-kinase were selected as the candidate genes for PM resistance. Three kompetitive allele-specific PCR (KASP) markers, *Kasp_PMR_2AS* for *QPm.caas-2AS*, *Kasp_PMR_4BL* for *QPm.caas-4BL*, and *Kasp_PMR_6BS* for *QPm.caas-6BS*, were developed, and their genetic effects were validated in a natural population including 100 cultivars.

**Discussion:** These findings will offer valuable QTLs and available KASP markers to enhance wheat marker-assisted breeding for PM resistance.”

The corrected paragraph appears below:

“**Introduction:** Fusarium crown rot (FCR) is a severe soil-borne disease that affects wheat globally and leads to significant yield reductions. Identifying the loci associated with resistance to FCR and developing corresponding markers are essential for the breeding of resistant wheat varieties.

**Methods:** In this study, we evaluated the resistance to FCR in a recombinant inbred line (RIL) population originating from Gaocheng 8901 and Zhoumai 16 across four environments. The RILs and their parents were genotyped using a wheat 90K single-nucleotide polymorphism (SNP) array.

**Results:** We identified a total of five quantitative trait loci (QTLs) related to FCR resistance: *QFCR.caas-3AL*, *QFCR.caas-3DL*, *QFCR.caas-5BL*, *QFCR.caas-6BS*, and *QFCR.caas-7DS*. These QTLs accounted for 4.6% to 12.8% of the phenotypic variance. Notably, *QFCR.caas-5BL* and *QFCR.caas-6BS* had been previously detected, whereas *QFCR.caas-3AL*, *QFCR.caas-3DL*, and *QFCR.caas-7DS* are novel loci. The favorable alleles of *QFCR.caas-3DL* and *QFCR.caas-5BL* were contributed by Zhoumai 16, while the favorable alleles for *QFCR.caas-3AL*, *QFCR.caas-6BS*, and *QFCR.caas-7DS* originated from Gaocheng 8901. Additionally, this study identified seven candidate genes that encode disease resistance proteins, the BTB/POZ domains, peroxidase activity, and leucine-rich repeat receptor-like protein kinase. Furthermore, we developed and validated two kompetitive allele-specific PCR (KASP) markers, *Kasp_3AL_FCR* (*QFCR.caas-3AL*) and *Kasp_5BL_FCR* (*QFCR.caas-5BL*), in a natural population of 202 wheat varieties.

**Discussion**: This study contributes new genetic insights and provides new stable loci and available KASP markers for breeding to enhance FCR resistance in common wheat.”

In the published article, there was an error in **Figure 3** as published. The leftmost box plot did not include the different letters to mark significant differences. The corrected **Figure 3** appears below.

**Figure 3:**
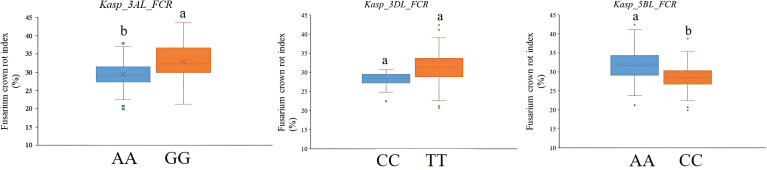
Validation of *Kasp_3AL_FCR, Kasp_3DL_FCR*, and *Kasp_5BL_FCR* in the panel of 202 wheat cultivars from the Huang-Huai River Valleys region. Different letters indicate significant differences at the *P <*0.05 level.

The authors apologize for these errors and state that this does not change the scientific conclusions of the article in any way. The original article has been updated.

