# Correction: Genome-wide linkage mapping of Fusarium crown rot in common wheat (*Triticum aestivum* L.)

**DOI:** 10.3389/fpls.2026.1936848

**Published:** 2026-07-22

**Authors:** Faji Li, Can Guo, Qi Zhao, Weie Wen, Shengnan Zhai, Xinyou Cao, Cheng Liu, Dungong Cheng, Jun Guo, Yan Zi, Aifeng Liu, Jianmin Song, Jianjun Liu, Jindong Liu, Haosheng Li

**Affiliations:** 1 Crop Research Institute, National Engineering Laboratory for Wheat and Maize, National Key Laboratory of Wheat Improvement, Key Laboratory of Wheat Biology and Genetic Improvement in the Northern Yellow-Huai Rivers Valley of Ministry of Agriculture and Rural Affairs, Shandong Academy of Agricultural Sciences, Jinan, China; 2 Shangqiu Academy of Agriculture and Forestry Sciences, Shangqiu, China; 3 Collage of Life Science, Yantai University, Yantai, China; 4 Department of Cell Biology, Zunyi Medical University, Zunyi, Guizhou, China; 5 Institute of Crop Sciences, National Wheat Improvement Center, Chinese Academy of Agricultural Sciences (CAAS), Beijing, China

**Keywords:** common wheat, Fusarium crown rot (FCR), molecular marker-assisted selection, quantitative trait Loci (QTL), kompetitive allele-specific PCR (KASP)

An incorrect number was provided for “Natural Science Foundation of Shandong Province”. The correct number is “(ZR2023MC205)”.

The original version of this article has been updated.

