# Supplementary material for: Genome-wide linkage mapping of Fusarium crown rot in common wheat (Triticum aestivum L.)
Source: Front Plant Sci. 2024 Nov 1;15:1457437. doi: 10.3389/fpls.2024.1457437 (PMC11563792; doi:10.3389/fpls.2024.1457437)
Supplement: Supplementary file 3 [file Table3.docx]

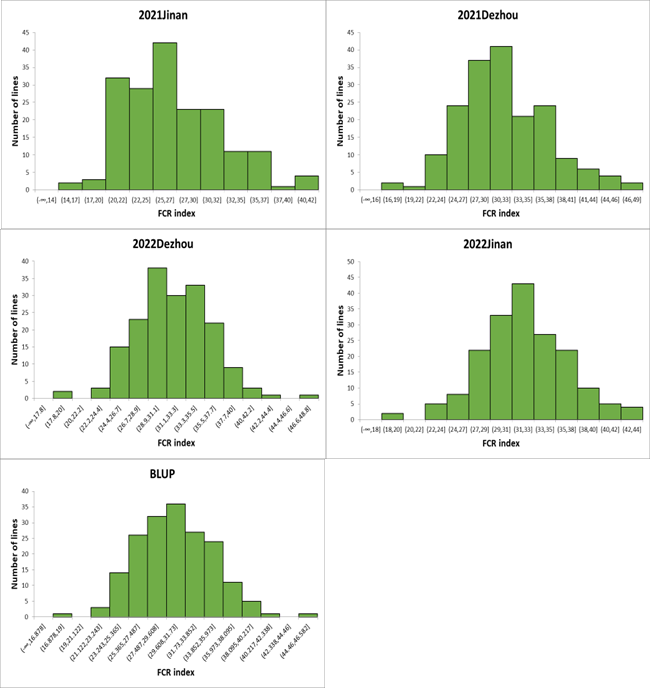


**Fig. S1** Frequency distributions for FCR index in the Gaocheng 8901/zhoumai16 RIL population in four environments and BLUE values.
