# Supplementary figures and images for: Genome-wide linkage mapping of Fusarium crown rot in common wheat (Triticum aestivum L.)
Source: Front Plant Sci. 2024 Nov 1;15:1457437. doi: 10.3389/fpls.2024.1457437 (PMC11563792; doi:10.3389/fpls.2024.1457437)

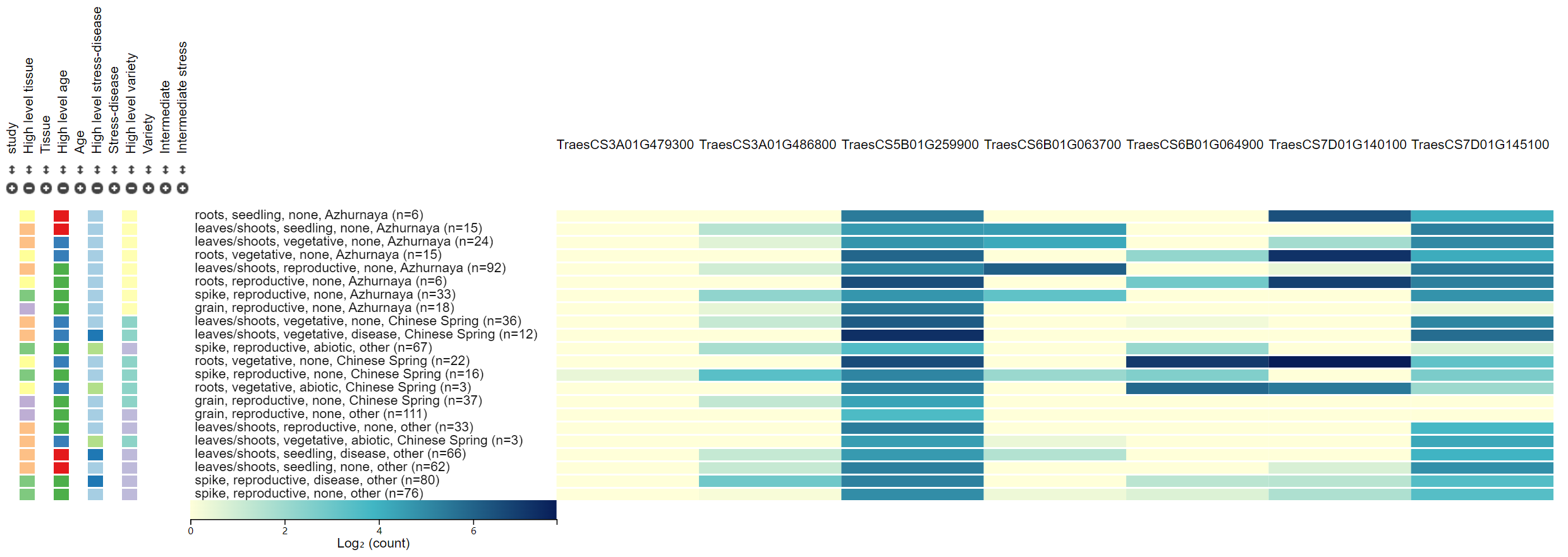


**Fig. S2** The expression pattern of the 7 candidate genes in the expVIP database

Supplement: Supplementary file 4 [file Table4.docx]
